# Young population awareness of head and neck cancer's (HNC) risk factors, symptoms and prognosis – a pilot survey

**DOI:** 10.1186/1753-6561-9-S7-A5

**Published:** 2015-10-27

**Authors:** Anna Krentowska, Anna Skoneczny, Elzbieta Sierko, Ewa Sierko

**Affiliations:** 1 Students' Scientific Association in the Department of Oncology, Medical University, Bialystok, Poland; 2 Department of Oncology, Medical University, Bialystok, Poland

## Background

HNC is the sixth most common type of cancer in Europe, with over 150,000 new cases in 2012. It is usually recognized at an advanced clinical stage, when the survival rate is remarkably lower, compared to patients diagnosed early. Main risk factors, tobacco and alcohol use, are usually present in young people, thus health education in this group is crucial. The aim of our study was to evaluate the level of HNC awareness among young population in North-Eastern Poland.

## Methods

An anonymous questionnaire about HNC was conducted among 1665 people in the age of 18-35 years. Population consisted mainly of high school and university students. Respondents were asked about HNC risk factors, symptoms and prognosis.

## Results

Eighty five percent of respondents had heard about HNC. The main source of information was the Internet (57%). Students of medical universities considered smoking (92%), alcohol (59%) and HPV infection (53%) main risk factors; other respondents indicated smoking (67%), stress (34%) and excessive sunbathing (32%). Almost all students knew that smoking causes lung cancer, whereas much less respondents realised that this risk factor causes also HNC (89% - laryngeal, 77% - oral, 76% - oropharyngeal cancer). As much as ¼ of the young people were unaware of the first symptoms of HNC. Medical students indicated chronic hoarseness (78%), chronic sore throat (68%) and difficulty or pain while swallowing (68%), while almost ½ of the students of technical universities didn't know any of the symptoms. Alarmingly, 5% of medical students and 10% of the remaining respondents would see the doctor only when the symptoms made everyday functioning impossible. Only 62% of the study population knew that early diagnosis of HNC is associated with 90% chance of cure.

## Conclusions

Awareness about HNC among young people is disturbingly low. Students of non-medical schools and universities have little knowledge on risk factors and first symptoms of HNC. Extending the population further and including low-educated young people in the study would be reasonable. Increasing the number of educational campaigns would lead to earlier presentation, diagnosis and treatment.

